# Evidence of Zika virus horizontal and vertical transmission in *Aedes albopictus* from Spain but not infectious virus in saliva of the progeny

**DOI:** 10.1080/22221751.2020.1830718

**Published:** 2020-10-17

**Authors:** Ana I. Nuñez, Sandra Talavera, Lotty Birnberg, Raquel Rivas, Núria Pujol, Marta Verdún, Carles Aranda, Miguel Berdugo, Núria Busquets

**Affiliations:** a IRTA, Centre de Recerca en Sanitat Animal (CReSA, IRTA-UAB), Campus de la Universitat Autònoma de Barcelona, Bellaterra, 08193, Spain; b Consell Comarcal del Baix Llobregat, Servei de Control de Mosquits, Barcelona, Spain; c Institut de Biología evolutiva de Barcelona, Universidad Pompeu Fabra-CSIC, Dr. Aigüader 88, Barcelona, 08003, Spain

**Keywords:** Zika virus, *Aedes albopictus*, vector competence, vertical transmission

## Abstract

*Aedes albopictus* mosquitoes have been experimentally demonstrated to be a competent vector for Zika virus (ZIKV) in different countries, but there are still some gaps related to the importance of *Ae. albopictus* in ZIKV transmission. Recent studies on Spanish *Ae. albopictus* populations showed controversial results for ZIKV transmission and no studies have been performed yet to detect infectious ZIKV in saliva of progeny of infected female mosquitoes. Herein, the horizontal transmission (HT) and vertical transmission (VT) of ZIKV in field-collected *Ae. albopictus* mosquitoes from Spain were evaluated for ZIKV strains (African I and Asian lineages) to better estimate the risk of ZIKV transmission by *Ae. albopictus*. The two field-collected *Ae. albopictus* populations assayed were infected by all tested ZIKV strains, however differences in terms of vector competence were detected depending on strain-population combination. Moreover, a higher susceptibility to the African I lineage strain than to the Asian lineage strain was observed in both mosquito populations. On the other hand, VT was demonstrated for both ZIKV lineages, detecting the virus in both males and females of the progeny of infected females, although importantly ZIKV dissemination and transmission were not detected in the infected females from the offspring. The results of the present study demonstrate that Spanish *Ae. albopictus* populations could sustain virus transmission in case of ZIKV introduction, but VT would play a poor role in the ZIKV epidemiology. Overall, our results provide helpful information to health authorities to establish efficient surveillance and vector control programmes for ZIKV.

## Introduction

Zika virus (ZIKV) is an emergent arthropod-borne virus that belongs to *Flavivirus* genus within *Flaviviridae* family [1]. It was isolated for the first time in 1947 from a rhesus monkey in the Zika forest (Uganda) [2], and afterwards in an *Aedes africanus* pool from the same localization in 1948 [3]. Hereafter, since the 1960s, ZIKV was sporadically found outside the African continent and until 2007, when ZIKV emerged in the Yap Island (Micronesia) [4] causing the first large outbreak. Then, it spread to the Pacific islands in 2013 [5] and reached the American continent in 2015 [6], where it finally affected more than 45 countries [1,7]. All phylogenetic analyses performed by now, confirm that ZIKV was originated in the African continent and then spread to Asia, Pacific Islands and the Americas [8]. Currently, ZIKV is classified into three lineages: Asian, African I, and African II [9]. The Asian lineage caused the spread of ZIKV into the Americas [1]. Due to the recent outbreaks in the Americas and since it has been demonstrated to cause microcephaly in neonates, the virus has become a threat to the public health [1].

Zika virus is maintained in a sylvatic zoonotic cycle among primatophilic mosquitoes and non-human primates [10]. The epidemic cycle occurs between humans and anthropophilic mosquitoes maintaining the virus in the urban sites [1]. The viral infection in humans generally occurs by the bite of infected females, but sexual [11] and perinatal [12] ZIKV transmission have also been reported in humans. In this sense, ZIKV vertical transmission (VT) has also been shown in mosquitoes; ZIKV was detected in adult mosquitoes, which came from reared field-eggs collected of some mosquitoes from *Aedes* genus [1,13,14]. In addition, ZIKV was detected in field collected adult males of *Aedes furcifer* [13], *Aedes aegypti* and *Culex quinquefasciatus* mosquitoes indicating venereal or vertical transmission in the field [10,15]. Moreover, ZIKV VT has also been experimentally demonstrated in *Ae. aegypti* [16–18] and *Ae. albopictus* mosquitoes [17]; and venereal transmission, in *Ae. aegypti* mosquitoes [19]. Zika virus VT has been suggested as an important mechanism to maintain the virus in the environment during hostile conditions [16]. However, the epidemiological importance of ZIKV VT in mosquitoes in nature and the transmission by the progeny of infected females is still unknown since no studies have been performed yet to detect infectious ZIKV in saliva of progeny of infected females.

Recently, numerous *Aedes* mosquitoes from all continents have been shown to be experimentally susceptible to transmit the virus, such as *Ae. vittatus*, *Aedes vexans* [1], *Aedes polynesiensis* [20] and *Ae. Albopictus* [21–25]*.* The Asian tiger mosquito species*, Ae. albopictus*, which was originated in the forests of Southeastern Asia, is widely distributed in tropical and subtropical regions. Nowadays, due to its global expansion, it has been catalogued as an invasive mosquito species [26]. This mosquito species, which is an anthropophilic and day-time feeder [1], is known to be a competent vector of several arboviruses, such as chikungunya virus (CHIKV), West Nile virus (WNV) or dengue virus (DENV) [27]. Its current expansion and establishment in Eastern Spain since 2004 [28] along the Mediterranean coast and Balearic Islands, and its anthropophilic feeding behaviour, threat seriously the public health of the country. It has been experimentally demonstrated that Mediterranean *Ae. albopictus* mosquitoes are competent vectors for ZIKV [22,25,29,30]. However, differences in vector competence (VC) among *Ae. albopictus* populations have been observed for ZIKV [31]. Moreover, recent studies with Spanish *Ae. albopictus* populations have shown controversial results in terms of ZIKV susceptibility and transmission [30,32,33]. Additionally, three cases of ZIKV autochthonous transmission have been reported in France [34], emphasizing the importance to determine if Spanish field-collected *Ae. albopictus* mosquito populations can transmit the virus in order to develop an efficient vector surveillance and control programmes by the competent authorities. The results of ZIKV transmission studies allow to decide which mosquito species are relevant in the ZIKV epidemiology so that they must be considered a target for ZIKV surveillance and reduced to avoid the spread of the virus and mitigate its locally circulation. For all the above-mentioned reasons, in the present study, we evaluated: (i) the VC of two field-collected Spanish *Ae. albopictus* mosquito populations for the African I and Asian ZIKV lineages, and (ii) VT and viral transmission of the offspring of intrathoracically inoculated *Ae. albopictus* females with the African I and Asian ZIKV lineages to better understand ZIKV VT and its importance in the ZIKV epidemiology. These studies provide relevant and helpful information to health authorities to design the strategies of the ZIKV surveillance programmes.

## Material and methods

### Mosquito rearing

Eggs from two populations of *Ae. albopictus* from El Prat de Llobregat and Rubí (Catalonia, Spain [Fig S1]), were collected to evaluate the VC of ZIKV. In addition, F0-F1 of field-collected *Ae. albopictus* females from Rubí municipality were also used for the VT assays. Larvae were reared in trays containing dechlorinated water supplemented with yeast (Gayelord- Hauser, Saint-Genis-Laval, France) until the adult stage. Emerging adults were maintained in the laboratory under controlled environmental conditions: 28°C, 80% of relative humidity and a light/dark cycle of 12:12 h. Adult mosquitoes were provided with 10% sucrose solution *ad libitum*.

### Zika virus strains

Two ZIKV strains (Dakar and Martinique) were used in the present study to evaluate the VC of local mosquito populations. The Dakar strain (UVE/ZIKV/1984/SN/Dakar ArD 411662, African I lineage, passage 4, Genbank reference: KU955592) was isolated from an *Aedes taylori* mosquito species in Senegal in 1984 and the Martinique strain (MRS_OPY_Martinique_ PaRi_2015, Asian lineage, passage 3, Genbank reference: KU647676) was isolated from human serum in 2015. Both ZIKV strains were passaged one more time in our laboratory and titrated in Vero cells (ATCC, ref. CCL-81) to obtain the 50% tissue culture infective dose per mL (TCID_50_/ml).

### Oral infection of mosquitoes for vector competence assays

The VC experiment was performed using the same protocol recently reported by Vazeille et al. [25]. Briefly, a total of 879 *Ae. albopictus* adult females of 7–9 days old, respectively, were starved for 24 h and artificially fed with washed rabbit erythrocytes mixed with adenosine 5’-triphosphate (ATP) (5 × 10^−3^ M) (Sigma-Aldrich, St. Louis, MO) and containing ZIKV from a frozen viral stock (final concentration: 7 log_10_ TCDI _50_/mL of Dakar and Martinique strains) (Table 1). The infectious blood was provided to mosquitoes by an artificial Hemotek feeding system set at 37.5°C ± 0.5 (Discovery Workshop, Accrington, UK). Females were fed during 30 min. Then, blood-engorged females were anaesthetized on ice, selected, and maintained in groups of 10 in cardboard cages (Watkins & Doncaster, Leominster, UK) under the above-mentioned rearing conditions. Throughout the experiment, mosquitoes were maintained with 10% sucrose solution *ad libitum*. After a period of 7, 14, and 21 days post exposure (dpe) to infectious blood females were anaesthetized on ice. The legs and wings were dissected and the saliva was extracted using a tip with 5 μl of FCS (fetal calf serum), where the proboscis was introduced during 30 min, and collected in 45 μl of Dulbecco’s modified Eagle’s medium (DMEM) (Gibco, MA, USA). The head, body and saliva samples were stored in 300 μl of DMEM supplemented with 2% FCS and 1 X anti–anti (Gibco Life Technologies 100 ml 15240-062 100X) at −80°C until virus isolation. To evaluate the VC four rates were assessed: (i) the infection rate (IR), which represents the proportion of females with infected body among tested females; (ii) the disseminated infection (DIR), that refers to the proportion of females with infected head among females with infected body; (iii) the transmission rate (TR), which is the ratio between females with infectious saliva among females with disseminated infection; and lastly (iv), the transmission efficiency (TE) that is the percentage of females with infectious saliva among the total number of mosquito females tested [31]. The experimental infections were carried out at *Institut de Recerca i Tecnologia Agroalimentàries- Centre de Recerca en Sanitat Animal* (IRTA-CReSA) BLS3 facilities.

**Table 1.** Summary of vector competence assays.

				Number of mosquitoes tested per time point			
Mosquito species tested	Population	ZIKV strains tested	Titer of ZIKV (TCID 50/ml)	7 dpe^a^	14 dpe^a^	21 dpe^a^	Total
*Ae. albopictus*	El Prat de Llobregat	Martinique	7 log_10_ TCID_50_/ml	20	20	17	57
	El Prat de Llobregat	Dak84	7 log_10_ TCID_50_/ml	11	13	12	36
*Ae. albopictus*	Rubí	Martinique	7 log_10_ TCID_50_/ml	28	27	22	77
	Rubí	Dak84	7 log_10_ TCID_50_/ml	28	27	25	80

^a^ Dpe: days post-exposure.

### Intrathoracical ZIKV inoculation of mosquitoes for vertical transmission assays

*Aedes albopictus* (F0-F1) females from Rubí municipality were used for the VT assays. A total of 69, 29, and 38 females of 5–7 days old were intrathoracically-inoculated with the Dakar (African I lineage) and Martinique (Asian lineage) strains at 7.5 log_10_TCID_50_/ml and with the Martinique strain at 8.2 log_10_TCID_50_/ml, respectively. All intrathoracically inoculated females were maintained at the rearing conditions mentioned above. All females were starved for 24 h and fed with fresh rabbit blood at 7- and 14-days post-inoculation (dpi) to obtain the eggs of the first (E1) and second (E2) ovipositions. The eggs were dried during 7 days at rearing conditions and then hatched for subsequent mosquito rearing. Finally, adult males from the offspring of the inoculated females were sacrificed at 7 days old and whole bodies were kept at −80°C. Meanwhile, adult females from the same generation were anaesthetized on ice and dissected at 14 days old to allow the virus reach salivary glands. Males were sacrificed earlier than females since they die before females and only infection of the bodies was evaluated. Females were dissected, legs and wings were detached from the body to allow saliva extraction and heads were also collected. All female mosquito samples (head, body and saliva samples) were stored at −80 °C until viral isolation following the same protocol described above. Finally, the filial infection rate (FIR) was used to estimate the proportion of infected progeny among all eggs produced by the inoculated females and the IR, DIR and TR of the progeny of the females inoculated with ZIKV were determined.

### ZIKV detection

The head and body samples obtained from the experimental infections were thawed and homogenized in 0.5 ml of DMEM at 30 Hz for 1 min using TissueLyser II (Qiagen GmbH, Stockach, Germany). Virus detection in head and body samples was performed by inoculating on 96 well plates with Vero CCL-81 cells, as described by Valleize et al. [25]. To summarize, Vero cells inoculated with the samples were maintained using DMEM, which was supplemented with 2% FCS and 1 X anti–anti, during seven days at 37 °C and 5% CO_2_. After this period, to observe the cytopathic effect (CPE), cells were stained with a 0.2% crystal violet solution (C.I. 42555, Merck KGaA, Darmstadt, Germany) and 2% of formaldehyde (Electron Microscopy Sciences, Hatfield, PA, USA) during 30 min. Saliva samples were titrated on six well plates with Vero CCL-81 cells using a method described previously by Arias-Goeta et al. [35]. After seven days of incubation under agarose overlay, the agar was removed, and the cells were stained with crystal violet solution for 30 min. Viral titers from saliva samples were expressed as plaque forming units per volume (PFUs/ml). The saliva samples from the VT experiment were titrated in 96-well plates in a Vero cell monolayer and incubated for seven days at same conditions above-mentioned until cytopathic effect evaluation.

### Statistical analyses

The statistical differences between mosquito populations, virus strains and dpe were analysed using a generalized linear model with a binomial family distribution (logistic regression). In this model, we analysed both nominal effect and all factor interactions. Time post exposure was treated as a factor and not as a continuous variable. Post-hoc analyses to compare between mosquito populations among ZIKV lineages at each time point were performed with a *χ*^2^ test using Monte Carlo permutations to determine the *p*-value. The statistical analyses were conducted using R 3.6 [36]. The *p*-value < 0.05 was considered statistically significant. The TR and TE were not analysed by the logistic regression model due to the lack of variance in the response variable driven by an excess of 0.

## Results

### Evidence that vector competence of field-collected *Ae. albopictus* populations for Zika virus depends on strain-population combination

**Figure 1.**
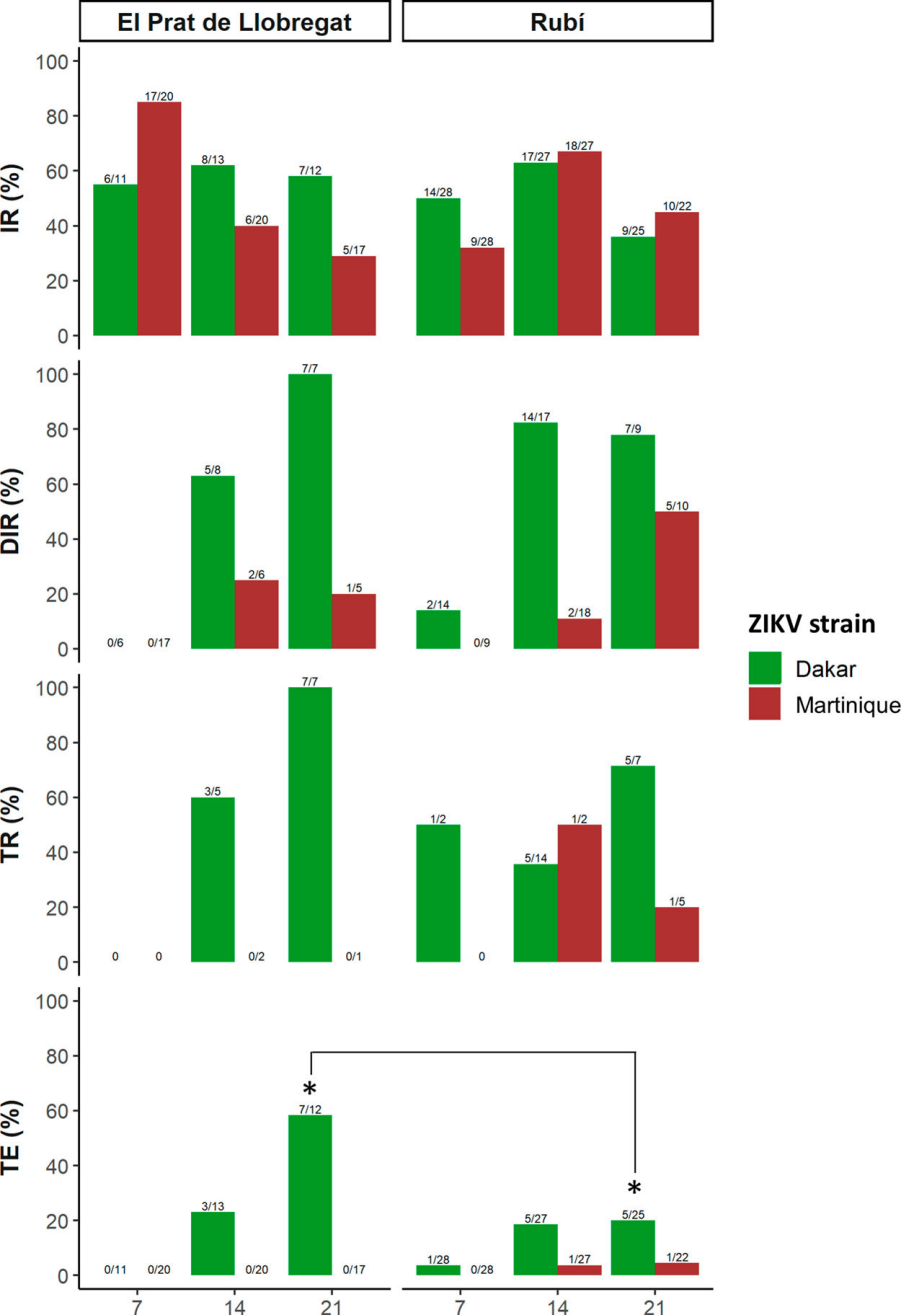
Infection, dissemination and transmission rates and transmission efficiency of two field-collected *Ae. albopictus* populations exposed to Dakar and Martinique ZIKV strains, which are represented with red and green colours, respectively. The * in the figures shows the statically significant results between populations. IR: infection rate; DIR: dissemination rate; TR: transmission rate; TE: transmission efficiency.

The feeding rates were 58.5% (277/473) and 27.3% (111/406) for *Ae. albopictus* from Rubí and El Prat de Llobregat populations*,* respectively. Infected bodies with ZIKV were detected from both field-collected *Ae. albopictus* populations exposed orally to infectious blood with both ZIKV strains tested at all-time points (7, 14, and 21 dpe) (Figure 1, Table S1). At 7 dpe, an early dissemination and transmission of the African I lineage (Dakar strain) could only be observed in the Rubi population. However, the proportion of females with infectious saliva among the total tested females (TE) was higher at 21 dpe in El Prat de Llobregat population than in Rubí population (*p *= 0.02) for Dakar strain.

Regarding Martinique strain, Rubí population was able to transmit it at 14 and 21 dpe; being the TE slightly higher at 21 dpe (TE = 4.5%) than at 14 dpe (TE = 3.7%) (Figure 1). No viral particles of the Martinique strain were isolated in mosquito saliva samples from El Prat de Llobregat population probably may be due to the small sample size.

### Spanish *Ae. albopictus* populations were more competent to the African I lineage than to the Asian ZIKV lineage

When viral strains were compared we observed that viral dissemination was higher for Dakar strain (African I lineage) than for Martinique strain (Asian lineage) in both assayed mosquito populations along the infection (*p *= 1.18E-06) (Figure 1, Table 2, and S1). These results indicated that the midgut infection barrier was similar for both ZIKV strains throughout the infection, but the viral dissemination varied depending on the virus strain. The midgut escape barrier was stronger for the Martinique strain (Asian lineage) than for the Dakar strain (African I lineage) in both populations due to its low dissemination at all tested time-points.

**Table 2.** Comparison of disseminated infection rates between mosquito population, dpe and viral strains using a generalized linear model with a binomial family distribution (logistic regression).

	DIR		
	d.f.	*χ*^2^	*p* value
**Dpe**	2	28.551	6.31E-07***
**Strain**	1	23.6072	1.18E-06***
**Population**	1	0.0524	0.8189
Dpe*Strain	2	2.482	0.2891
Dpe*Population	2	1.7889	0.4088
Strain*Population	1	1.4643	0.2262
Dpe*Strain*Population	2	4.2258	0.1209

***p-value <0.0001; Dpe: days post-exposure; d.f.: degrees of freedom.

With regards to the El Prat de Llobregat population, the TE (positive saliva samples among all blood feed mosquitoes) for Dakar strain (African I lineage) showed 23% and 58.3% at 14 and 21 dpe, respectively, while Martinique strain (Asian lineage) was not detected in any saliva sample. Moreover, in the Rubí population, Dakar strain (African I lineage) presented 18.5% and 20% of positive saliva among the assayed mosquito samples at 14 and 21 dpe, while Martinique strain (Asian lineage) presented 3.7% and 4.5% at 14 and 21 dpe, respectively. Furthermore, Dakar strain (African I lineage) TE for both populations was higher at 21 dpe than at 14 dpe.

**Figure 2.**
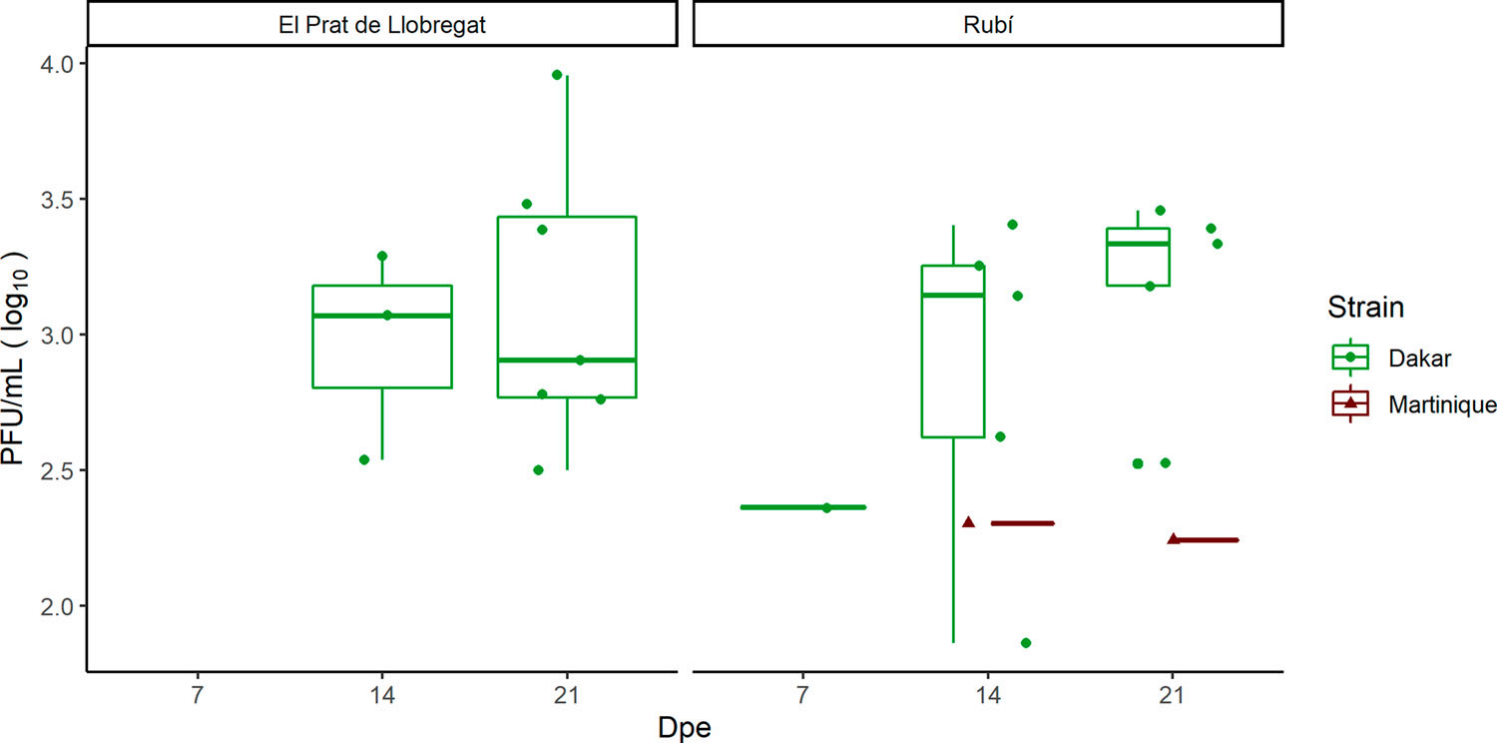
ZIKV loads in saliva (Plaque forming units [PFU/mL]) of infected female mosquitoes from two field-collected *Ae. albopictus* mosquito populations (El Prat del Llobregat and Rubí) exposed to Dakar and Martinique ZIKV strains. *Dpe: days post-exposure to the virus.

Regarding to viral titers in saliva of those female mosquitoes that were able to expectorate ZIKV within the saliva, both *Ae. albopictus* populations which were tested, showed higher viral titers in saliva samples for the Dakar strain (mean = 1712.4 PFU/ml) than for the Martinique strain (mean = 186.8 PFU/ml), in which the size of the positive samples was low (2). The viral loads in the saliva of positive mosquitoes are shown in the Figure 2.

### Vertical transmission was detected in *Ae. albopictus* but the infected females of the progeny were not able to disseminate and transmit ZIKV

**Figure 3.**
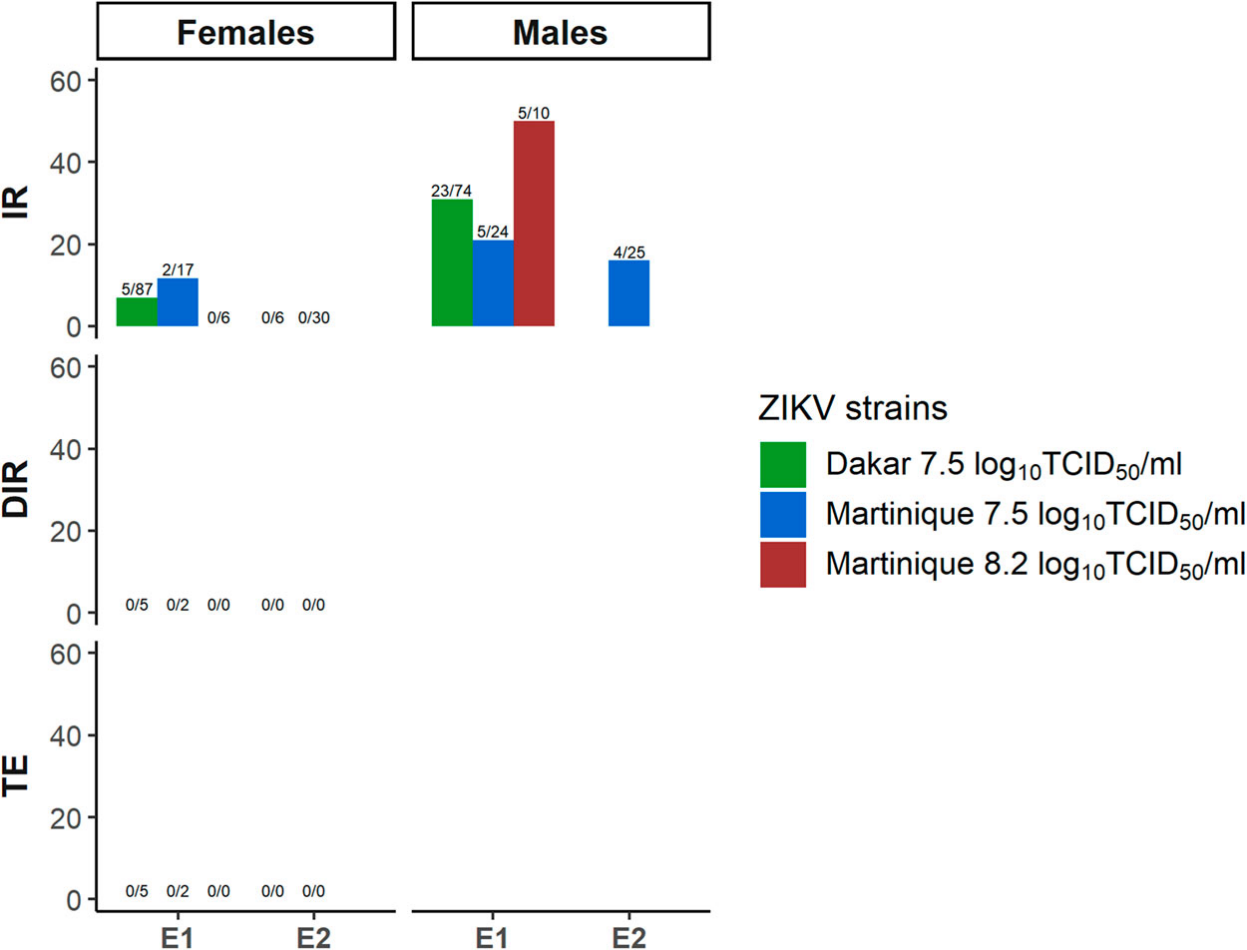
Infection and dissemination rates and transmission efficiency of the progeny of infected females from of one field-collected *Ae. albopictus* population (Rubí) for Dakar and Martinique ZIKV strains. IR: infection rate; DIR: dissemination rate; TE: transmission efficiency.

Infection and dissemination of the inoculated females were confirmed at 7 dpi. Zika virus was detected in bodies of both males and females from the progeny of the E1 of inoculated females for both tested ZIKV lineages (Table 3). In addition, infected bodies were detected in males from the E2 of inoculated females for the Asian lineage. All heads and saliva samples from infected females of the progeny resulted negative for both ZIKV lineages (Table S2 and Figure 3).

**Table 3.** Number of eggs obtained from ZIKV inoculated females during the first and second gonotrophic cycle and filial infection rates (including males and females).

Specie	Generation	Strains	Viral doses	FEF females	Egg E1	Filial infection rate	Egg E2	Filial infection rate
***Ae. albopictus***	F0-F1	Dak84	7.5 log_10_ TCID_50_/ml	34	559	1: 19.9	322	<1: 322 (0%)
***Ae. albopictus***	F0-F1	Martinique	7.5 log_10_ TCID_50_/ml	19	272	1: 38.8	94	1: 23.5
***Ae. albopictus***	F1	Martinique	8.2 log_10_ TCID_50_/ml	12	140	1: 28	133	N.D.

Note: FEF: full-engorged inoculated females; E1: first gonotrophic cycle; E2: second gonotrophic cycle; N.D.: No data.

## Discussion

Due to the ZIKV outbreak in 2015 in Brazil, the amount of scientific research about this virus has recently increased because of its medical importance. Consequently, the number of VC studies for ZIKV has also augmented in the last years since virus transmission via mosquito bite plays a crucial role to determine the risk of introduction of a pathogenic agent in a non-endemic country. European *Ae. albopictus* mosquitoes have been demonstrated to be competent vectors for ZIKV although showing heterogeneous results [22,25,29,30,37]. Our present study also shows the infection susceptibility of two field-collected *Ae. albopictus* populations from Spain for two ZIKV lineages (African I and Asian). Our results are particularly in agreement with Vazeille et al. [25], which is easily comparable since they performed the experiment using the same protocol (viral dose, viral strains, environmental conditions, procedure) established in the frame of the European ZIKAlliance project. Vazeille et al. [25] demonstrated that *Ae. albopictus* populations from France (Montpellier and Corsica) were more susceptible to Dakar strain (African I lineage) than to Martinique and Cambodia strains (Asian lineage), which agrees with our findings. Both tested populations in the present study showed significant differences in transmission at 21 dpe for the African I lineage, as also previously presented by Vazeille et al. [25]. Moreover, our results showed an early transmission of the African I lineage at 7 dpe in *Ae. albopictus* population from Rubí as also previously demonstrated by Vazeille et al. [25] for the African I lineage in an *Ae. albopictus* population from France and Gutiérrez-López et al. [10] for the Asian lineage in a Spanish *Ae. albopictus* population. This early transmission, which means a relatively short extrinsic incubation period, reported for *Ae. albopictus* mosquitoes could increase their vectorial capacity (commonly defined as the number of infectious bites a host receives daily [38]). A high vectorial capacity may increase the risk of infection in case of ZIKV introduction into the Mediterranean countries since the infected mosquito females could transmit the virus at least from 7 dpe. Besides, our results showed differences in terms of VC for ZIKV between the two tested mosquito populations. These differences could also be observed when we compared our results with those obtained for French *Ae. albopictus* mosquitoes. They seem to be better vectors for Dakar strain (African I lineage) than Spanish populations at 14 dpe. The TR obtained was around 80% in both assayed French mosquito populations, and our transmission results were 60% and 35% in El Prat de Llobregat and in Rubí population respectively; although the transmission was similar at 21 dpe for all French (around 80%) and Spanish mosquito populations (100% and 71%, in El Prat de Llobregat and Rubí, respectively). In addition, Spanish *Ae. albopictus* were less competent for the Asian lineage than French *Ae. albopictus*, where the TR was around 60% for Cambodia strain and 40% for Martinique strain. However, in our study, transmission was only detected in Rubí population for Martinique strain at 14 and 21 dpe in a low number of tested mosquitoes (TR was 50% and 20%, respectively). Regarding the viral titer in saliva samples, Rubí mosquito population showed higher viral titer for the African I lineage than for the Asian lineage as previously showed Vazeille et al. for French mosquito populations, besides the viral titers obtained in both studies were similar [25]. European results show VC differences between *Ae. albopictus* mosquito populations as it was previously pointed out in American mosquito populations [31] and as other studies have previously described for DENV [39].

The vector competence of Spanish *Ae. albopictus* populations remained controversial considering that two recent studies [32,33] showed that the virus was not detected in the expectorated saliva in contrast to another recent study [30], which tested an *Ae. albopictus* population from the same region of Spain (Barcelona province) and showed ZIKV transmission. Our results, which agree with the latest study, suggest that these contradictory results could be explained by at least four main reasons. Firstly, the rearing temperature, which is an extrinsic factor that affects direct and indirectly the *Ae. albopictus* susceptibility to arbovirus [23,40,41]. The environmental factors in which mosquitoes were reared, may have played a crucial role for ZIKV transmission of Spanish *Ae. albopictus* populations. The VC study performed by Hernández-Triana e*t al*. (2019), where the rearing temperature was low (20°C/25°C [night/day]), did not detect transmission for ZIKV, in contrast with the results of other VC studies that showed ZIKV transmission, where the experiments were performed under temperature conditions that ranged from 26°C to 28°C. Secondly, the viral concentration used to artificially feed the females could have also influenced the VC of Spanish *Ae. albopictus* mosquitoes for ZIKV. The low titer (1.8 × 10^6^ PFU/ml; *cf.* the above-mentioned European mosquito studies and even our assay) used in the study of González *et al.* (2019) could explain their negative results in terms of ZIKV transmission. Thirdly, the VC of *Ae. albopictus* females for ZIKV depends on mosquito population and virus lineage specific interactions [42] as previously demonstrated in *Ae. aegypti* and *Ae. albopictus* mosquitoes for DENV and ZIKV [31,39]. Lastly, besides mosquito-virus interaction, the number of generations of laboratory colony influences VC [25,43]. It is a possible explanation of why the laboratory colony (from 2009) used in the VC experiment by Hernández-Triana *et al*. (2019) has not been able to transmit ZIKV. It is important to take into account that the laboratory colonies would not be the best option to perform VC experiments since the long-term colonization may cause genotypic and phenotypic changes in mosquitoes [43].

To know how ZIKV could be maintained in nature during periods without active vectors and available vertebrate hosts is essential to understand the ZIKV ecology. In this regard, the VT has been suggested as an alternative mechanism to preserve the virus in the environment [16]. Arboviral VT takes place through two mechanisms: transovarial transmission and trans-ovum transmission [44–46]. It is thought that the VT of flaviviruses (*e.g*. DENV) generally occurs by trans-ovum transmission [17,47]. The ZIKV VT has been previously reported for *Ae. albopictus* [17,48] and other species in the field [13,49], but the viral transmission in the progeny of infected females had not been tested until now. Our results confirmed VT, since infected males and females of the E1 progeny of intrathoracically inoculated *Ae. albopictus* for both African I and Asian lineages were detected. These results agree with a recent study performed in *Ae. albopictus* mosquitoes [48], in which they detected the Asian lineage of ZIKV in males and females from the first oviposition progeny. A recent study conducted with orally infected *Ae. aegypti* mosquitoes with Asian ZIKV lineage has shown that VT moderately decreased with gonotrophic cycles [18], which would agree with our results in *Ae. albopictus*, since we found higher prevalence in E1 progeny than in the E2 progeny. However, our results contrast with previous results obtained by Thangamani et al. [16] and Phumee et al., where VT in *Ae. albopictus* was not demonstrated for the Asian lineage. These contrasting results could be explained because of the viral load within the infected mosquitoes, since they used a lower viral dose and the specimens were orally infected [50], and as it was discussed above, viral dose may affect ZIKV infection in *Ae. albopictus* mosquitoes. To our knowledge, this is the first time that the viral transmission in the infected progeny has been evaluated for ZIKV; the obtained results indicated that all infected females of the progeny were not able to disseminate the virus through hemolymph and transmit the virus, suggesting that ZIKV VT in *Ae. albopictus* might be not very relevant for ZIKV epidemiology.

Arboviruses (e.g. La Crosse virus and dengue virus) could be amplified in a mosquito population by a venereal transmission mechanism [51,52]. In this case, male mosquitoes would obtain arboviruses by VT from an infected female and thereby, could transmit the virus horizontally to a non-infected female during mating as demonstrated in experimental studies for La Crosse virus in *Aedes triseriatus* [51]. After that, the females may develop infected oocytes, generating infected progeny [16,51,53]. In this regard, the ZIKV detection in *Ae. albopictus* males in our experiment and in mosquito males from the field reported in previous studies [13,49] suggests that they could play a role in the preservation of ZIKV in nature due to its polygamous behaviour [54] as it was previously suggested for DENV infection [46]. Venereal transmission for ZIKV was first described in *Ae. aegypti* [19]. However, there is still a lack of knowledge about the epidemiological relevance of venereal transmission in ZIKV transmission by *Ae. albopictus*. Further studies are required to confirm the role of males in the transmission cycle of ZIKV.

To sum up, our study demonstrated that both evaluated Spanish *Ae. albopictus* populations were competent vectors for the African I and Asian ZIKV lineages since infectious ZIKV was isolated from saliva of experimentally infected mosquitoes, although with some differences in terms of viral susceptibility and transmission depending on mosquito population and virus strain. Therefore, *Ae. albopictus* established in the Barcelona province (Spain) may sustain virus transmission when the ZIKV is introduced. Moreover, VT of ZIKV was proved in Spanish *Ae. albopictus* but the obtained results suggest that viral transmission from females of the progeny of ZIKV infected females might be not highly frequent and further studies on ZIKV transmission are needed to elucidate the role of VT in ZIKV epidemiology. Overall, our results provide supportive information to the health authorities to apply efficient surveillance and vector control programmes for ZIKV.

## Supplementary Material

Fig_S1._Map_of_the_sampling_sites.tif

Supp_Table_S2.docx

Supp_Table_S1.docx
