# Supplementary figures and images for: Evidence of Zika virus horizontal and vertical transmission in Aedes albopictus from Spain but not infectious virus in saliva of the progeny
Source: Emerg Microbes Infect. 2020 Oct 17;9(1):2236–44. doi: 10.1080/22221751.2020.1830718 (PMC7594878; doi:10.1080/22221751.2020.1830718)

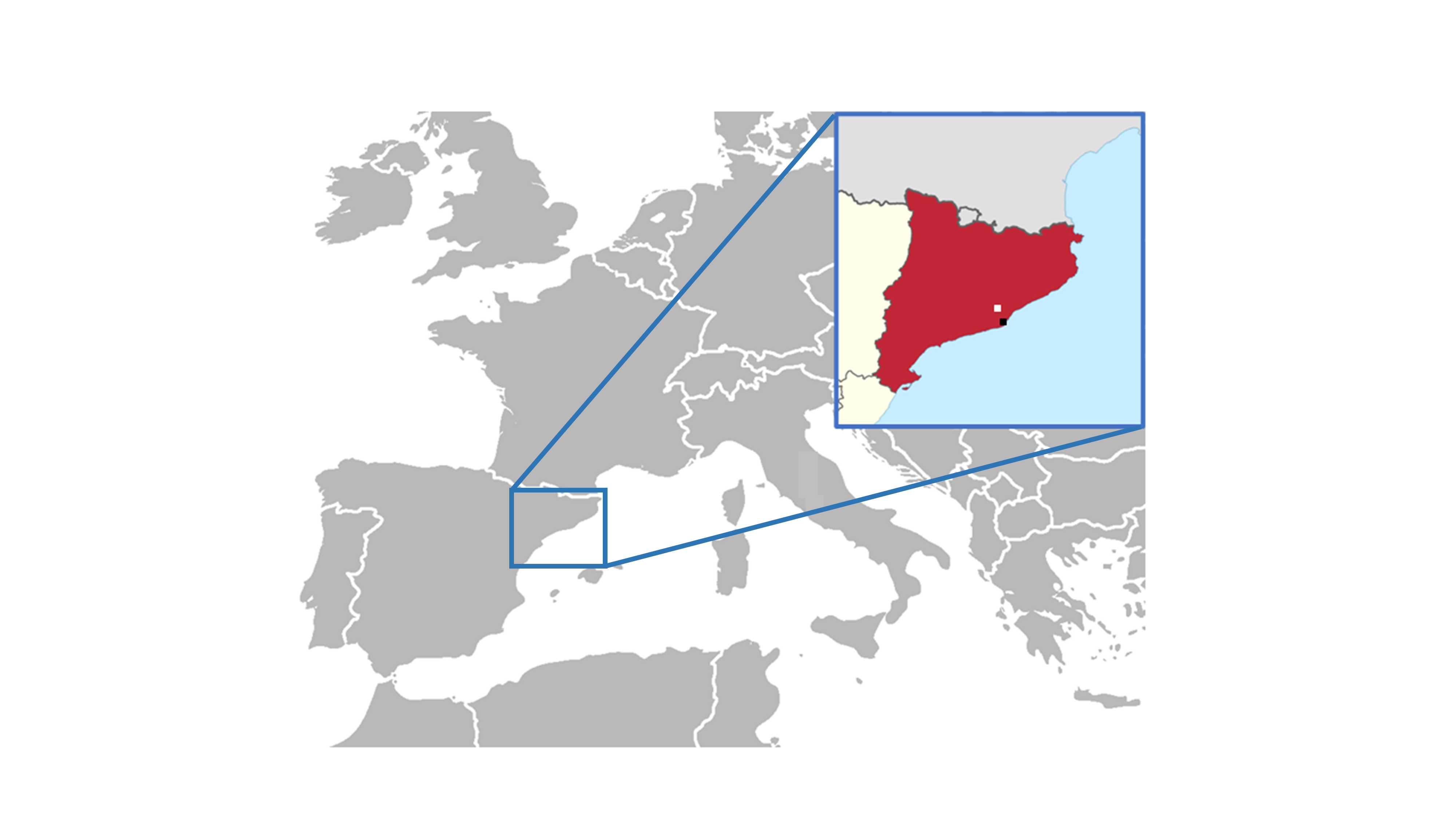

Supplement: Fig_S1._Map_of_the_sampling_sites.tif [file TEMI_A_1830718_SM0889.tif]
