# Supplementary material for: Evidence of Zika virus horizontal and vertical transmission in Aedes albopictus from Spain but not infectious virus in saliva of the progeny
Source: Emerg Microbes Infect. 2020 Oct 17;9(1):2236–44. doi: 10.1080/22221751.2020.1830718 (PMC7594878; doi:10.1080/22221751.2020.1830718)
Supplement: Supp_Table_S2.docx [file TEMI_A_1830718_SM0888.docx]

**Supplemental material: Table S2.** Infection, disseminated infection and transmission rates of the offspring of the *Ae. albopictus* females from Rubí intrathoracically inoculated with Dak84 and Martinique ZIKV strains.

|  |  |  | **Males** | |  | **Females** | | | |  |
| --- | --- | --- | --- | --- | --- | --- | --- | --- | --- | --- |
|  |  |  | **E1** | **E2** | **E1** | |  | **E2** | |  |
| **Species** | **Strain** | **Viral dose** | **IR (%)** | **IR (%)** | **IR (%)** | **DIR (%)** | **TR (%)** | **IR (%)** | **DIR (%)** | **TR (%)** |
| ***Ae. albopictus*** | Dak84 | 7.5 log_10_ TCID_50_/ml | 23/74 (31%) | 0/6 (0%) | 5/87 (5.7%) | 0/5 (0%) | 0/0 (0%) | 0/6 (0%) | 0/0 (0%) | 0/0 (0%) |
|  | Martinique | 7.5 log_10_ TCID_50_/ml | 5/24 (20.8%) | 4/25 (16%) | 2/17 (11.7%) | 0/2 (0%) | 0/0 (0%) | 0/30 (0%) | 0/0 (0%) | 0/0 (0%) |
|  | Martinique | 8.2 log_10_ TCID_50_/ml | 5/10 (50%) | 0/4 (0%) | 0/6 (0%) | 0/0 (0%) | 0/0 (0%) | - | - | - |

IR= infection rate; DIR= disseminated infection rate; TR: transmission rate; E1: first gonotrophic cycle; E2: second gonotrophic cycle; -: No data.
