# Supplementary material for: Evidence of Zika virus horizontal and vertical transmission in Aedes albopictus from Spain but not infectious virus in saliva of the progeny
Source: Emerg Microbes Infect. 2020 Oct 17;9(1):2236–44. doi: 10.1080/22221751.2020.1830718 (PMC7594878; doi:10.1080/22221751.2020.1830718)
Supplement: Supp_Table_S1.docx [file TEMI_A_1830718_SM0887.docx]

**Supplemental material: Table S1**. Infection, dissemination and transmission rates of two field *Ae. albopictus* populations exposed to Dak84 and Martinique ZIKV strains.

| **Specie** | **Population** | **ZIKV strain** | **Dpe** | | | | | | | | | |
| --- | --- | --- | --- | --- | --- | --- | --- | --- | --- | --- | --- | --- |
|  |  |  | **7 dpe** | | | **14 dpe** | | | **21 dpe** | | | |
|  |  |  | **IR (%)** | **DIR (%)** | **TR (%)** | **IR (%)** | **DIR (%)** | **TR (%)** | **IR (%)** | **DIR (%)** | **TR (%)** |  |
| *Ae. albopictus* | El Prat de Llobregat | Martinique | 17/20 (85%) | 0/17 (0%) | - | 8/20 (40%) | 2/8 (25%) | 0/2 (0%) | 5/17 (29.4%) | 1/5 (20%) | 0/1 (0%) |  |
|  |  | Dak84 | 6/11 (54.5%) | 0/6 (0%) | - | 8/13 (61.5%) | 5/8 (62.5%) | 3/5 (60%) | 7/12 (58.3%) | 7/12 (58.3%) | 7/7 (100%) |  |
| *Ae. albopictus* | Rubí | Martinique | 9/28 (32.12%) | 0/9 (0%) | - | 18/27 (66.6%) | 2/18 (11.1%) | 1/2 (50%) | 10/22 (45.4%) | 5/10 (50%) | 1/5 (20%) |  |
|  |  | Dak84 | 14/28 (50%) | 2/14 (14.2%) | 1/2 (50%) | 17/27 (70%) | 14/17 (82.3%) | 5/14 (35.7%) | 9/25 (36%) | 7/9 (77.7%) | 5/7 (71.4%) |  |

IR= infection rate; DIR= dissemination rate. TR= transmission rate; Dpe: days post-exposure.
